# A phase 2 randomized, double-blind trial of ART-001, a selective PI3Kα inhibitor, for the treatment of slow-flow vascular malformations

**DOI:** 10.1186/s13023-025-03564-z

**Published:** 2025-02-10

**Authors:** Michio Ozeki, Akira Tanaka, Kanako Kuniyeda, Taiki Nozaki, Akihiro Fujino, Tadashi Nomura, Naoto Uemura, Souichi Suenobu, Noriko Aramaki-Hattori, Ayato Hayashi, Aiko Kato, Hiro Kiyosue, Kotaro Imagawa, Munetomo Nagao, Fumiaki Shimizu, Junko Ochi, Saya Horiuchi, Tetsuji Ohyama, Haruhi Ando, Hiroshi Nagabukuro

**Affiliations:** 1 Department of Pediatrics, Graduate School of Medicine, Gifu University, Gifu, Japan; 2 ARTham Therapeutics Inc., Yokohama, Kanagawa Japan; 3 Department of Clinical Pharmacology and Therapeutics, Faculty of Medicine, Oita University, Oita, Japan; 4 Department of Radiology, St. Luke’s International Hospital, Tokyo, Japan; 5 Division of Pediatric Surgery National Center for Child Health and Development, Tokyo, Japan; 6 Department of Plastic Surgery, Kobe University Graduate School of Medicine, Kobe, Hyogo Japan; 7 Division of General Pediatrics and Emergency Medicine, Department of Pediatrics, Oita University Faculty of Medicine, Oita, Japan; 8 Department of Plastic and Reconstructive Surgery, Keio University School of Medicine, Tokyo, Japan; 9 Department of Plastic and Reconstructive Surgery, Juntendo University Urayasu Hospital, Chiba, Japan; 10 Department of Plastic and Reconstructive Surgery, Faculty of Medicine, Oita University, Oita, Japan; 11 Department of Radiology, Faculty of Medicine, Oita University, Oita, Japan; 12 Department of Plastic Surgery, Tokai University, Sagamihara, Kanagawa Japan; 13 Department of Plastic and Reconstructive Surgery, Faculty of Medicine, Tohoku University, Sendai, Miyagi Japan; 14 Department of Radiology, Suita Tokushukai Hospital, Osaka, Japan; 15 Biostatistics Center, Kurume University, Fukuoka, Japan; 16 Present Address: SFG SCIENCES Inc., 24-8 Yamashita-cho, Naka-ku, Yokohama, Kanagawa 231-0023 Japan; 17 Present Address: Department of Radiology, Keio University School of Medicine, Tokyo, Japan; 18 Present Address: Department of Pediatric Surgery, Keio University School of Medicine, Tokyo, Japan; 19 National Hospital Organization Nishibeppu National Hospital, Oita, Japan; 20 Present Address: Department of Plastic and Reconstructive Surgery, Yokohama City University School of Medicine, Yokohama, Kanagawa Japan; 21 Present Address: Department of Diagnostic Radiology, Faculty of Life Science, Kumamoto University, Kumamoto, Japan; 22 Present Address: Department of Radiology, University of Iowa Hospitals and Clinics, Iowa City, Iowa USA

**Keywords:** Phase 2 study, Vascular malformations, PI3Kα, Pharmacotherapy, And drug development

## Abstract

**Background:**

In patients with slow-flow vascular malformations (SFVMs) including venous malformations (VM), lymphatic malformations (LM) or Klippel–Trenaunay Syndrome (KTS), somatic gain-of-function mutations in genes encoding phosphatidyl inositol 3-kinase alpha (PI3Kα, gene name *PIK3CA*) have been identified. A phase 2 study was conducted with the patients to assess the efficacy and safety of ART-001 (serabelisib), an orally available selective PI3Kα inhibitor.

**Methods:**

This is a multicenter, randomized, double-blind, proof-of-concept, phase 2 trial. Eligible participants were patients aged 2 years and older, diagnosed either with VM, LM or KTS. Participants were administered either 50 or 100 mg of ART-001 for 24 weeks. The primary endpoint was the response rate defined as the proportion of participants who achieved ≥ 20% reduction in lesion volume at week 24. Secondary endpoints include safety, pharmacokinetics, pain, and quality of life scores.

**Results:**

Thirty-five patients (median age: 14 years old; VM, n = 17, KTS, n = 13 and LM, n = 5) were randomly assigned and received treatment (50 mg, n = 17 and 100 mg, n = 18). ART-001 showed a response rate: 29.4% (95% confidence interval 10.3–56.0%) at 50 mg and 33.3% (13.3–59.0%) at 100 mg. Mean lesion volume reductions at 50 mg and 100 mg were − 2.3% (95% CI − 14.3 to 9.6%) and − 12.6% (− 25.3 to 0.06%), respectively. No drug-related serious adverse events were observed. Treatment-emergent adverse events were generally mild to moderate and transient. Pharmacokinetic profiles were similar between pediatric and adolescent/adult patients except for lower C_trough_ levels in pediatric patients.

**Conclusion:**

ART-001 was effective and well-tolerated in patients with SFVMs. These results support the further development of ART-001 in SFVMs and other PIK3CA-related overgrowth syndromes to confirm clinical benefits and long-term safety.

*Trial registration*: Japan Registry of Clinical Trial, jRCT2071210027. Registered May 25 2021,https://jrct.niph.go.jp/en-latest-detail/jRCT2071210027

**Supplementary Information:**

The online version contains supplementary material available at 10.1186/s13023-025-03564-z.

## Background

Venous malformation (VM), lymphatic malformation (LM), and Klippel–Trenauney syndrome (KTS) are rare diseases with a prevalence of 1–2 in 1,000,000, 1 in 6000 to 1 in 16,000 live births, and 1 in 1,000,000 live births, respectively [1–3]. They are congenital slow-flow vascular malformations (SFVMs) and are usually present at birth and often symptomatic, causing pain and disfigurement. The primary treatments of SFVMs are resection, sclerotherapy/embolization, and laser therapy [3, 4]. As most of them are invasive and many patients are not applicable to these primary treatment options, because of the large size of the lesions and/or the risk of functional loss due to surgery, there are significant clinical needs for pharmacotherapies.

Recent studies revealed that the phosphatidylinositol 3-kinase alpha (PI3Kα)/protein kinase B (AKT)/ mammalian target of rapamycin (mTOR) pathway plays a key role in the pathogenesis of SFVMs [4]. Somatic gain of function mutations of PI3Kα and its up- and down-stream kinases were identified in the lesions from the patients with SFVMs [5–8], suggesting activation of the pathway is an etiological mechanism of the diseases. Indeed, pharmacological inhibition of this pathway has been demonstrated its efficacy in SFVMs. For example, sirolimus, a mTOR inhibitor, improved the symptoms including pain and quality of life (QOL) in patients with SFVMs in multiple clinical trials [9–12]. Ji et al. also reported sirolimus decreased the lesion volume in patients with VM, LM, KTS, and combined vascular malformations [13]. Alpelisib, a selective PI3Kα inhibitor originally approved for breast cancer, is now granted accelerated approval by FDA for the treatment of PIK3CA-related overgrowth spectrum (PROS) including KTS based on a real-world data from a compassionate use clinical study [14]. Alpelisib demonstrated that 27% of patients experienced ≥ 20% reduction from baseline in the target lesion volume at week 24.

ART-001 (serabelisib, also known as TAK-117/MLN1117/INK1117) is a selective PI3Kα inhibitor originally developed for advanced solid tumor [15]. ART-001 showed inhibition of angiogenesis and antitumor effects on human tumor cells with gain of function mutations in the *PIK3CA* gene, which encodes PI3Kα. In the recent phase 1 study, ART-001 with a novel dry syrup formulation was safe and well-tolerated up to 100 mg once-daily dose with a favorable pharmacokinetics (PK) profile in healthy volunteers [16].

For the purpose of designing a phase 2 clinical trial of ART-001, we have recently conducted a multicenter prospective observational study in patients with intractable VM and KTS to understand the natural course of the diseases without any interventions [17]. The study demonstrated that there was no statistically significant difference in the lesion volume, pain score and QOL score over 6 months without any intervention, scientifically and ethically supporting a phase 2 clinical trial without a placebo control group. Following the observational study, we conducted the phase 2 multicenter, randomized, double-blind, proof-of-concept trial of ART-001 in patients with VM, LM and KTS to evaluate the efficacy, safety, and PK of ART-001at 50 mg and 100 mg.

## Methods

### Post-hoc analysis of the prospective observational natural history study

To prepare this phase 2 study, we have recently conducted a multicenter prospective observational study in patients with intractable VM and KTS [17]. In the study, target lesion volume, pain score, QOL score and other parameters were measured before and after a 6-month observation period without any interventions for the diseases. Among 34 patients (VM n = 17, KTS n = 17) who completed the study, patients who had a history of infection within four weeks prior to the registration were removed, and the proportion of patients who achieved ≥ 20% reduction in lesion volume was calculated.

### Phase 2 study design

This is a multi-center, randomized, double-blind, proof-of-concept, phase 2 trial conducted from July 16, 2021 to July 28, 2022. In this study, the efficacy, PK and safety of ART-001 were evaluated in patients either with VM, LM, or KTS. The study was conducted in accordance with the study protocol, the Declaration of Helsinki, and the International Conference on Harmonization Guidance for Good Clinical Practice. The study was registered under jRCT2071210027.

### Subjects

The study included patients aged two and older, who had symptomatic and refractory VM, LM, or KTS with magnetic resonance (MR) images available and had not received any invasive treatment including sclerotherapy or surgical resection, and had not participated in any clinical trials within 12 weeks at obtaining consent. Once enrolled, MR images of the target lesion were assessed if the lesion volume was measurable. Patients with at least one measurable lesion were formally registered in this study. Exclusion criteria included a history of inflammatory infection requiring treatment of the target lesion within 4 weeks at obtaining consent and previous treatment with PI3Kα inhibitors or sirolimus within 12 weeks at obtaining consent.

### Study protocol and interventions

The participants were randomly assigned in a 1:1 ratio to receive 50 mg or 100 mg ART-001 orally once daily. Disease type was used as an allocation factor.

ART-001 was administered as dry syrup formulation after breakfast at 50 or 100 mg/day for 24 weeks. These doses of ART-001 were selected based on tolerability profile as well as blood glucose increase as a target engagement marker observed in the phase 1 study [16]. The dose was adjusted to body surface area in participants who were younger than 12 years old or whose weight was 40 kg or less. During the treatment period, study visits were performed every week for the first month and then every month.

### MR volumetry

All MR studies were performed on a 3.0-T or 1.5-T unit. Axial T1-weighted, fat-saturated (FS) T2-weighted, and coronal short tau inversion recovery (STIR) MR images were obtained at screening period (baseline), week 12 and week 24. The entire lesion volumes were extracted slice-by-slice from axial FS-T2 weighted MR images by two board-certified radiologists of the Japanese College of Radiology independently using a three-dimension volumetric segmentation function in OsiriX MD (Pixmeo, Inc, Switzerland), according to the prior study [17]. The average value of the results of them was adopted.

### Outcomes and measures

The primary endpoint was the response rate at week 24, defined as the proportion of participants who achieved ≥ 20% reduction from baseline in target lesion volume determined by MR volumetry. Secondary endpoints include the response rate at week 12, and changes in target lesion volume, pain score evaluated with VAS, QOL, and Performance Status. For QOL, the following questionnaires were used: Pediatric Quality of Life Inventory (PedsQL) 4.0 Generic core scale (2–4 years, parent proxy report; 5–18 years, patient report and parent proxy report; 18–25 years; patient report) [18–21] and 36-item short form health survey version 2 (SF-36v2, 26 years and older) [22–24]. In patients aged 2 to 4, the parent proxy report was considered as the patient report. Performance status was evaluated by using either Lansky play-performance scale (< 17 years old) [25] or Karnofsky performance scale (17 years and older).

### PK

The steady-state PK of repeated oral administration of ART-001 were evaluated. Blood samples were collected at pre-dose and 1–4 h after dosing on Day 29 and one day (20–29 h) after the last dose on Day 169. If allowed, the full PK analyses were performed in pediatric patients (≤ 12 years old). In the full PK analyses, blood samples were collected at pre-dose and 1, 2, 4, 6, 8, and 24 h after ART-001 administration. Plasma samples were analyzed for ART-001 using a validated liquid chromatography tandem mass spectrometry method at Sekisui Medical. The lower limit of quantification was 10 ng/mL.

### Safety assessments

Safety was assessed at regular intervals as mentioned above throughout the study and included monitoring of adverse events (AEs), vital signs, clinical laboratory tests, and physical examinations. All AEs were graded mild, moderate, or severe in intensity by the investigator. The frequencies of AEs were summarized using the Medical Dictionary for Regulatory Activities (version 23.1) preferred term. The safety analysis population includes all randomized participants with ART-001 administration (n = 35).

### Statistical analysis

Assuming an expected response rate of 50% with ART-001, α = 0.05 (two-tailed) and 1-β = 0.9, the required number of participants was calculated using 12.5% (2/16) as the threshold, which was obtained during the course of the observational study, resulting in a requirement of 15 participants. The expected discontinuation or dropout rate in this study was estimated to be about 10%, and the final number of participants to be included in the study was set at 34 (n = 17/group).

Efficacy was analyzed in the full analysis set (FAS), which comprised all randomized participants with ART-001 administration and with any efficacy data. For the primary efficacy analysis, the response rate (proportion of patients who showed a ≥ 20% reduction in the target lesion volume) and their 95% CI were calculated and verified if the lower limit of the 95% CI is greater than the threshold of 12.5% for at least one dose. In addition, *p*-values (one-sided) were calculated when the threshold of 12.5% was the null hypothesis. The significance level for dose-specific evaluation was adjusted using Bonferroni's method. 95% CIs and *p*-values were calculated using the Clopper Pearson method. For secondary outcomes, the response rate at week 12 was analyzed with the same method used to analyze that at week 24. Changes in target lesion volume from baseline were evaluated by the one-sample *t*-test. Changes in scores of pains, QOL, and performance status from baseline were evaluated by the one-sample Wilcoxon test. A *p*-value < 0.05 was considered statistically significant. All statistical analyses were performed using SAS software, version 9.4 (SAS Institute Inc.).

## Results

### Post hoc analysis of the prospective observational study

To estimate the proportion of the participants who showed ≥ 20% reduction in the natural course of the diseases without any intervention, the post hoc analysis of the prospective observational study was conducted. Since it was suggested that the lesion volume was largely affected by the local infection [17], four participants with a history of infection within 4 weeks before obtaining consent were excluded from the analysis. As a result, there were two participants who showed ≥ 20% reduction among 30 participants (6.7%) in the natural course of the diseases without any interventions.

### Phase 2 study

**Fig. 1:**
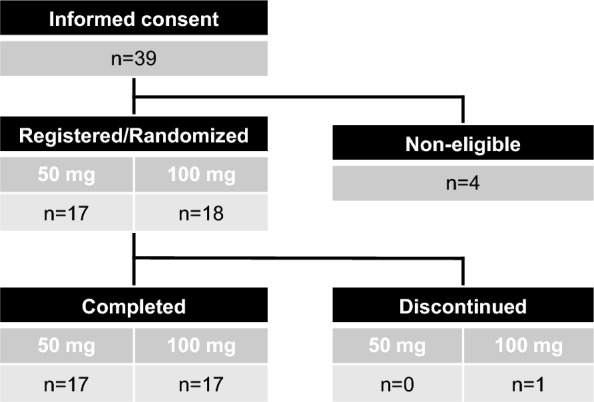
Participant enrollment consort diagram

Between July 2021 and July 2022, 39 participants were screened, and 35 eligible participants (age: 2–66 years old, VM, n = 17; KTS, n = 13; LM, n = 5) were enrolled and randomly assigned to either 50 mg or 100 mg group (50 mg n = 17 and 100 mg n = 18) (Fig. 1). Among them, 34 participants completed the study, and one patient in the 100 mg group discontinued due to the development of eczema. The compliance rates over 24 weeks were 96.7 ± 4.4% in the 50 mg group and 98.3 ± 2.9% in the 100 mg group. The participants’ demographics are summarized in Table 1. There were no significant differences between the two groups.

**Table 1:** Patient demographics

Characteristics	ART-001, 50 mg (n = 17)	ART-001, 100 mg (n = 18)
Sex		
Female, n (%)	9 (52.9)	12 (66.7)
Male, n (%)	8 (47.1)	6 (33.3)
Age at inclusion (years)		
Mean ± S.D. (min, max)	19.5 ± 17.4 (2, 66)	14.7 ± 10.3 (3, 43)
2–11, n (%)	7 (41.2)	7 (38.9)
12–17, n (%)	3 (17.6)	4 (22.2)
≥ 18, n (%)	7 (41.2)	7 (38.9)
Diagnosis, n (%)		
VM	9 (52.9)	8 (44.4)
LM	2 (11.8)	3 (16.7)
KTS	6 (35.3)	7 (38.9)
Height (cm), Mean ± S.D. (min, max)	139.3 ± 29.3 (85.0, 187,4)	137.2 ± 28.1 (91.0, 173.8)
Weight (kg), Mean ± S.D. (min, max)	42.4 ± 25.1 (10.9, 107.0)	39.3 ± 18.8 (13.1, 65.5)
Target lesion, n (%)		
Head and neck	6 (35.3)	6 (33.3%)
Upper limb	2 (11.8)	1 (5.6%)
Lower limb	8 (47.1)	11 (61.1%)
Trunk	1 (5.9)	0 (0.0)
Previous therapies*, n (%)		
Yes	12 (70.6)	15 (83.3)
Surgery	7 (41.2)	9 (50.0)
Sclerotherapy	8 (47.1)	9 (50.0)
Medication	5 (29.4)	7 (38.9)
Laser	4 (23.5)	4 (22.2)
Lesion volume at baseline (cm^3^),		
Mean ± S.D. (min, max)	803.7 ± 1080.6 (4.7, 3710.4)	719.4 ± 1013.7 (19.2, 4242.5)
Pain, n (%)		
No pain	4 (23.5%)	5 (27.8%)
Low (1–30)	6 (35.3%)	7 (38.9%)
Moderate (31–70)	4 (23.5%)	5 (27.8%)
High (71–100)	3 (17.6%)	1 (5.6%)
Lansky play-performance Scale, n (%)	n = 9	n = 8
100	1 (11.1)	0 (0.0)
90	3 (33.3)	6 (75.0)
80	4 (44.4)	2 (25.0)
70	1 (11.1)	0 (0.0)
≤ 60	0 (0.0)	0 (0.0)
Kranofsky Performance Status, n (%)	n = 8	n = 10
100	5 (62.5)	4 (40.0)
90	1 (12.5)	3 (30.0)
80	1 (12.5)	1 (10.0)
70	1 (12.5)	1 (10.0)
60	0 (0.0)	1 (10.0)
≤ 50	0 (0.0)	0 (0.0)

### Primary outcome

**Fig. 2:**
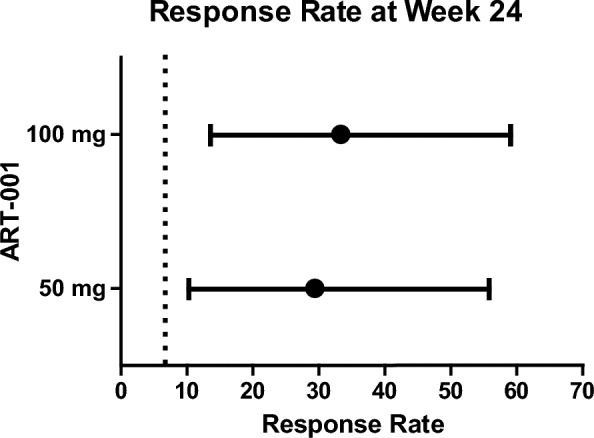
Response rates and the 95% CI at Week 24. Response rates in 50 mg group (n = 17) and 100 mg group (n = 18) were shown. The response rate was defined as the percentage of patients who showed more than a 20% reduction in the target lesion volume from baseline. The dotted line indicated 6.7%, which is the proportion of the participants who showed ≥ 20% reduction from baseline in lesion volume without any intervention in the six-month natural history study

ART-001 resulted in an increase in a response rate at week 24: 29.4% (n = 17, 95% Confidence Interval (CI) 10.3–56.0%) at 50 mg and 33.3% (n = 18, 13.3–59.0%) at 100 mg (Fig. 2). The lower limit of 95% CI of the response rate in the 100 mg group was greater than 12.5% (*p* = 0.0186), the pre-defined threshold, which was determined based on the interim results of the observational study. Following the study protocol, 12.5% was used for the statistical analysis as described in the Methods section. On the other hand, the lower limit of 95% CI of the response rates in both groups were greater than 6.7%, which is above described.

### Secondary outcomes

**Fig. 3:**
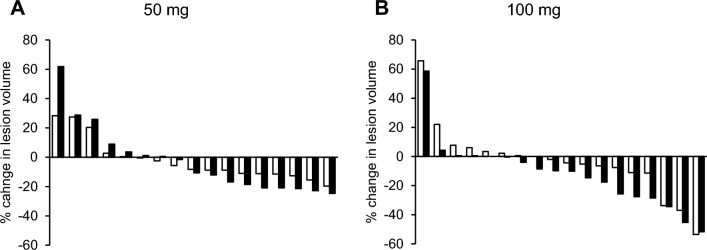
Waterfall plots of % changes in lesion volume. Data from each patient treated with ART-001 at 50 mg (**A**) and 100 mg (**B**) were plotted. The white bar indicates the data at week 12 and the black bar at week 24

The results of the secondary outcomes in efficacy are summarized in supplemental Table S1. The response rates at week 12 were 0.0% (95% CI 0.0–19.5) and 16.7% (3.6–41.4) in the 50 mg and 100 mg groups, respectively. The reduction in lesion volume in each participant at week 12 and 24 in the 50 mg and 100 mg groups are shown in Fig. 3. More than half of the patients showed a greater reduction at week 24 compared to that at week 12. The mean reduction of lesion volume at week 12 and 24 in the 100 mg group were − 3.80% and − 12.6%, respectively, and those in the 50 mg group were − 2.18% and − 2.34%, respectively. The absolute values of lesion size and their reduction are shown in Supplemental Table S2.

**Fig. 4:**
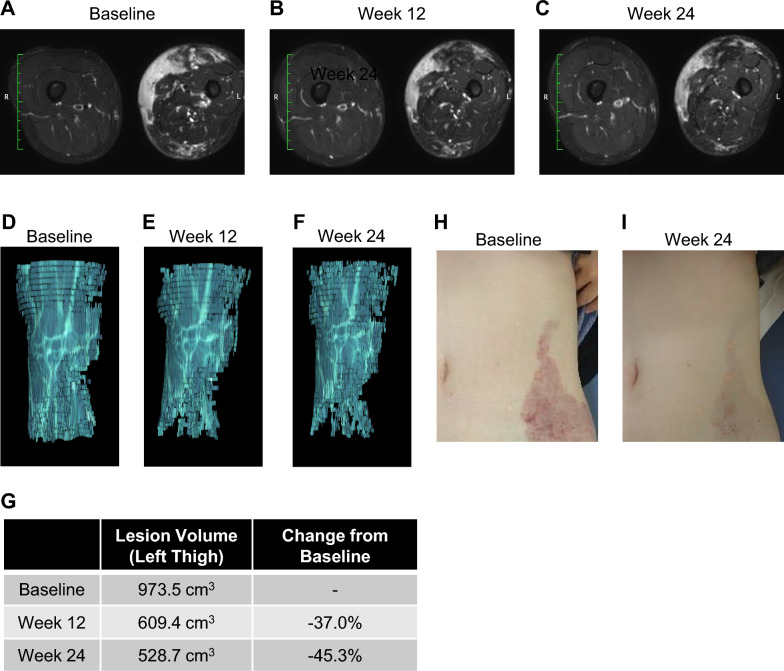
Representative radiological and clinical changes with ART-001. MR image (**A**–**C**), MR volumetry (**D**–**F**), and volume data (**G**) of the target lesion (left thigh), and clinical photograph (**H**, **I**) of an 18-year-old patient with KTS, who was treated with 100 mg ART-001. The data was taken at baseline (**A**, **D**, and **H**), week 12 (**B**, and **E**), and week 24 (**C**, **F**, and **I**)

Figure 4 shows the improvement of one patient with KTS treated with 100 mg of ART-001 according to the results of MR volumetry. She has a large lesion spread from her left abdomen to the lower limb. The target lesion was the left thigh. Compared to the baseline (973.5 cm^3^), the target lesion volume was reduced to 609.4 cm^3^ (37.0% reduction) and 528.7 cm^3^ (45.3% reduction) at week 12 and 24, respectively. At 24 weeks after administration, the patient showed marked improvement in skin lesion compared with pre-treatment.

**Fig. 5:**
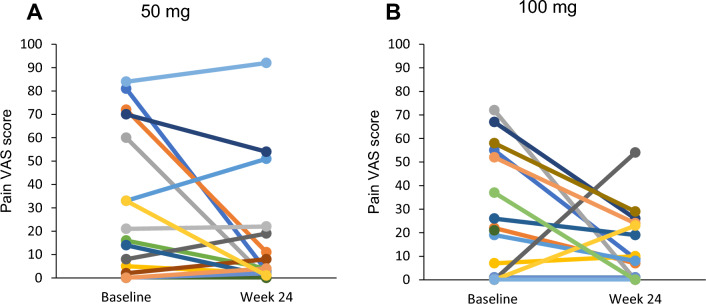
Pain VAS scores of each patient treated with ART-001. Data from each patient treated with ART-001 at 50 mg (**A**) and 100 mg (**B**) were plotted

The pain scores of each patient at baseline and at week 24 were plotted in Fig. 5. Many patients showed a decrease in the pain score. Thirteen patients (76.5%) in the 50 mg group, and 13 patients (72.2%) in the 100 mg group exhibited any symptoms (pain visual analog scale (VAS) score > 0) at baseline. The mean pain scores at baseline and at week 24 were 29.4 ± 31.4 and 16.1 ± 25.8 in the 50 mg group (n = 17) and 24.6 ± 26.6 (n = 18) and 12.4 ± 15.0 (n = 17) in the 100 mg group. The pain scores tended to decrease in both dosing groups, but there was no statistical significance.

**Fig. 6:**
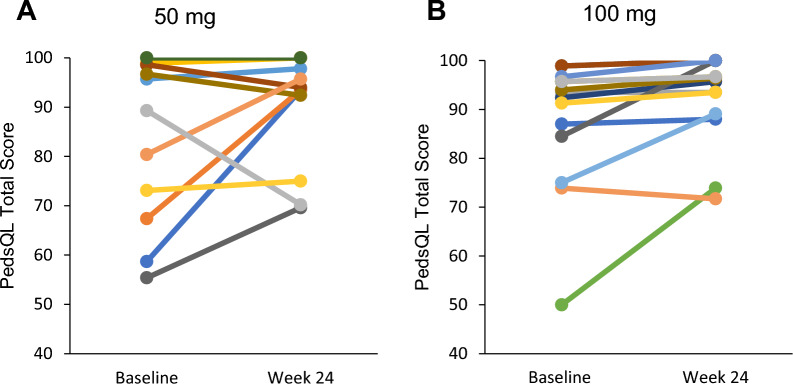
PedsQL total scores of each patient treated with ART-001. Data from each patient treated with ART-001 at 50 mg (**A**) and 100 mg (**B**) were plotted

QOL analyses were performed with PedsQL (2–25 years old) and SF-36 (≥ 26 years old). PedsQL total scores (self-report) of each patient were plotted (Fig. 6). Means of PedsQL total scores were 84.5 ± 16.9 (n = 12) and 86.5 ± 14.4 (n = 16) at baseline; and 90.1 ± 11.6 (n = 12) and 92.5 ± 9.2 (n = 14) at week 24 in the 50 mg and 100 mg groups, respectively. Significant improvement in change in PesQL total score from baseline was observed in the 100 mg group at week 24. The mean change of the PedsQL total score at week 24 from baseline in the 100 mg group was 5.21 (n = 14). However, there is no significant improvement observed in the subscales of the patient report and parent proxy report (Supplemental Table S1). Regarding SF-36, the sample numbers were too small to detect significant changes (50 mg group n = 5, and 100 mg group n = 2). There are no patients who showed a reduction in performance status and there are four patients who showed 10–20 points increase.

In addition to the effects measured as the endpoints, a reduction in redness (Fig. 4H, I) and softening of the affected area were observed in several patients.

### PK

**Fig. 7:**
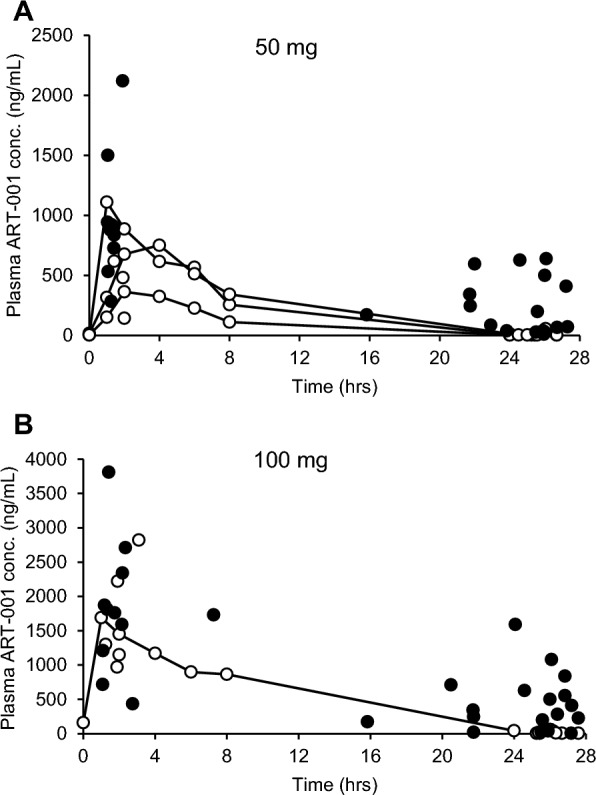
Steady-state plasma concentrations of ART-001 in patients. Data from patients in 50 mg (**A**) and 100 mg (**B**) were plotted. The open circle indicates the data from pediatric patients (< 12 years old) and the closed circle indicates the data from adolescent/adult patients (≥ 12 years old). Full PK was taken in 4 pediatric patients, 3 patients in the 50 mg group, and one patient in the 100 mg group (shown in open circles with connected lines)

The steady-state PK of repeated oral administration of ART-001 were evaluated (Fig. 7). The plasma concentrations at 1–4 h after dosing in the 50 mg group and 100 mg group were 830.83 ± 536.73 ng/mL (n = 12) and 1780.80 ± 893.17 ng/mL (n = 14), respectively. The mean plasma ART-001 concentrations at pre-dose (C_trough_) were 111.15 ± 207.34 ng/mL (n = 15) and 387.87 ± 498.96 ng/mL (n = 14) in the 50 mg and 100 mg groups, respectively. Full PK analysis was conducted with four pediatric patients (50 mg n = 3, 100 mg n = 1).

### Safety assessment

The incidence of treatment-emergent adverse events (TEAE) was similar in both dosing groups (50 mg, 82.4%; 100 mg, 77.8%) (Table 2). They were generally mild to moderate and transient. Details of TEAE are listed in Supplemental Table S3. No drug-related serious adverse events were observed in either group. Drug-related TEAE occurred in 7 (41.2%) and 7 (38.9%) patients at 50 mg and 100 mg, respectively. There was one patient who discontinued the administration of ART-001 by physician’s decision because of the grade 2 eczema. Drug-related TEAE occurred more frequently than 10% (2 patients) include nausea, stomatitis, alanine aminotransferase (ALT) increase, aspartate aminotransferase (AST) increase, and urine β2 microglobulin increase. Among them, ≥ Grade 3 drug-related TEAE were ALT increase (n = 3) and AST increase (n = 1), which was observed in three patients. The dosing of ART-001 was interrupted for two patients: one showed Grade 3 ALT increase and Grade 4 AST increase, and the other one showed Grade 1 ALT increase and Grade 1 AST increase. ART-001 administration were resumed after the ALT and AST increases were resolved.

**Table 2:** Adverse events

	ART-001, 50 mg (n = 17)	ART-001, 100 mg (n = 18)
Overall TEAEs, n (%)	14 (82.4)	14 (77.8)
Drug-related, n (%)	7 (41.2)	7 (38.9)
AEs leading to the discontinuation, n (%)		
Eczema	0 (0.0)	1 (5.6)
Serious AEs, n (%)	1 (5.9)	0 (0.0)
Drug-related, n (%)	0 (0.0)	0 (0.0)
≥ Grade 3 Drug-related TEAEs, n (%)	0 (0.0)	4 (22.2)
ALT increased	0 (0.0)	3 (16.7)
AST increased	0 (0.0)	1 (5.6)
Severe TEAEs, n (%)	0 (0.0)	0 (0.0)

Since hyperglycemia and serum creatinine increase were observed in the 300 mg once daily dosing group in the phase 1 study [16], the stopping criteria for them were set in this study. However, neither hyperglycemia nor serum creatinine increase were observed in the study (Supplemental Table S3).

## Discussion

This phase 2 clinical trial evaluated the efficacy, safety, and PK of a novel selective PI3Kα inhibitor ART-001 in patients with SFVMs. ART-001 at both 50 mg and 100 mg was well-tolerated and efficacious in reducing target lesion volume and improving QOL scores in the patients. There were higher response rates at week 24 in both 50 and 100 mg groups compared to the natural course of the diseases. Based on the post-hoc analysis of our previous observational study in patients with VM and KTS (LM not included), the proportion of patients who exhibited ≥ 20% reduction was 6.7%. Since there were no patients with more than 20% decrease in lesion volume for > 1 year in patients with LMs [26], we considered that 6.7% could be used for a natural history control in the current study. Response rates increased over 24-week period in both 50 mg and 100 mg groups, suggesting that the response rate may increase beyond 24 weeks, though long-term studies are needed to clarify the maximum effect and durability of the efficacy of ART-001.

Besides the subjective outcome associated with lesion volume, there is a significant improvement in the QOL score in patients aged 25 years and younger while QOL changes were not analyzed in the adult population due to the small sample size. It is necessary to accumulate the cases for further analysis on QOL.

The study demonstrated that repeated doses of ART-001 at 50 and 100 mg were safe and well-tolerated in patients with SFVMs. No death and no drug-related severe and serious AEs occurred in this study. One patient was discontinued due to eczema. Grade 3 and Grade 4 drug-related TEAE were ALT increase and AST increase. After dosing interruption in two patients, both ALT and AST levels reduced to the normal range.

As PI3Kα is located downstream of insulin receptors and plays an important role in glucose metabolism, hyperglycemia or blood glucose increase is an on-target effect of pharmacological inhibition of PI3Kα. Indeed, ART-001 dose-dependently caused hyperglycemia in its phase 1 study in healthy volunteers [16]. Hyperglycemia was also reported in PROS patients treated with alpelisib [14]. But in the current study, at the doses of 50 mg and 100 mg, that exhibited the least effect on fasting blood glucose levels in the phase 1 study, there was no hyperglycemia observed in patients, suggesting that PI3Kα inhibition required for the efficacy of ART-001 in SFVMs may not be that potent [15]. The mean steady-state plasma concentrations of ART-001 at 1–4 h after dosing were dose-proportionally increased between 50 mg (830.83 ng/mL) and 100 mg (1780.80 ng/mL) doses. The mean plasma concentration at 1–4 h of 100 mg dose was comparable to the mean maximum plasma concentration (C_max_) of 2128.3 ng/mL after repeated doses of 100 mg of ART-001 in healthy adults [16], confirming the consistency. Since the plasma concentrations at 1–4 h at both doses were comparable between pediatric and adolescent/adult patients, the ART-001 dose in pediatric patients was rightly adjusted using body surface area. C_trough_ levels of ART-001 in pediatric patients were lower than those in adolescent/adult patients, suggesting lower systemic exposure in pediatric patients while either the response rate or mean reduction of lesion volume was not lower in pediatric patients. Lower C_trough_ levels of ART-001 could be due to higher clearance of the drug in children, but its scientific rationale is not yet known.

The efficacy of alpelisib was evaluated using real-world data from EPIK-P1 (NCT04293723), a single-arm clinical study with patients with PROS [14]. In EPIK-P1 study patients with confirmed PIK3CA mutations were recruited. The response rate at week 24 was 27% (95% CI 14–44) and the mean change in target lesion was − 13.7% in EPIK-P1 study. In our study, the genetic test was optional and only 6 patients had the results. Before the Phase 2 study, the genetic analysis in patients with SFVMs with tissue samples collected during surgical procedures was conducted and it was demonstrated that 62.7% of the patients harbored pathogenic variants in genes involved in the PI3K signaling pathway [8]. Based on the results, we rationalized the recruitment of patients without genetic analysis. The effects of alpelisib were comparable to those observed with 100 mg ART-001, though the target population was different. The limitation of the current study includes the small sample size in each diagnostic and age group and long-term safety. It is necessary to accumulate the cases in future clinical trials.

## Conclusion

In this randomized phase 2 clinical trial, ART-001 achieved a significantly higher response rate compared to that observed in the natural disease course in 6 months treatment. Furthermore, ART-001 was safe and well-tolerated, and exhibited preferable PK in patients with SFVMs including the pediatric population. The results support the further development of ART-001 to confirm clinical benefits and long-term safety.

## Supplementary Information

Supplementary Material 1

## Data Availability

The data are available from the corresponding author upon reasonable request. Supporting data used for statistical analyses are provided in the Supporting Data Value file.

## Abbreviations

AEs: Adverse events
AKT: Protein kinase B
ALT: Alanine aminotransferase
AST: Aspartate aminotransferase
CI: Confidence interval
C_max_: Maximum plasma concentration
C_trough_: Plasma concentration at pre-dose
FDA: US Food and Drug Administration
FS-T2: Fat saturated T2
KTS: Klippel–Trenaunay syndrome
LM: Lymphatic malformations
MR: Magnetic resonance
mTOR: Mammalian target of rapamycin
PedsQL: Pediatric quality of life inventory
PI3Kα: Phosphatidyl inositol 3-kinase alpha
PIK3CA: Phosphatidyl inositol 3-kinase catalytic alpha
PK: Pharmacokinetics
QOL: Quality of life
SF-36: 36-Item short form health survey
SFVMs: Slow-flow vascular malformations
STIR: Short tau inversion recovery
TEAE: Treatment-emergent adverse events
VAS: Visual analog scale
VM: Venous malformations
