# Supplementary material for: A phase 2 randomized, double-blind trial of ART-001, a selective PI3Kα inhibitor, for the treatment of slow-flow vascular malformations
Source: Orphanet J Rare Dis. 2025 Feb 10;20:64. doi: 10.1186/s13023-025-03564-z (PMC11812195; doi:10.1186/s13023-025-03564-z)
Supplement: Supplementary file 1 — Supplementary Material 1 [file 13023_2025_3564_MOESM1_ESM.docx]

Supplemental Materials

| **Supplemental Table S1. Secondary Outcomes** | | | | | | |
| --- | --- | --- | --- | --- | --- | --- |
|  | **n** | **50 mg** | **p** | **n** | **100 mg** | **p** |
| Response Rate (%) |  |  |  |  |  |  |
| 12 week (95% CI) | 17 | 0.0 (0.0-19.5) | 0.1033 | 18 | 16.7 (3.6-41.4) | 0.3949 |
| Changes in lesion volume, Mean ± S.D. |  |  |  |  |  |  |
| Week 12 | 17 | -2.18 ± 14.42 | 0.5428 | 17 | -3.80 ± 25.54 | 0.5478 |
| Week 24 | 17 | -2.34 ± 23.28 | 0.6839 | 17 | -12.60 ± 24.63 | 0.0510 |
| Changes in Pain Score, Mean ± S.D. |  |  |  |  |  |  |
| Week 4 | 17 | -8.7 ± 16.1 | 0.0349 | 17 | -9.0 ±21.0 | 0.1445 |
| Week 12 | 17 | -8.2 ±17.5 | 0.1682 | 17 | -9.1 ± 20.9 | 0.1240 |
| Week 24 | 17 | -13.2 ± 28.1 | 0.1876 | 17 | -12.4 ± 29.5 | 0.0923 |
| Changes in QOL Score, Mean ± S.D. |  |  |  |  |  |  |
| PedsQL (2-25 years old, self-report) |  |  |  |  |  |  |
| Total Score |  |  |  |  |  |  |
| Week 12 | 12 | 3.70 ± 9.53 | 0.1875 | 14 | 3.82 ± 7.99 | 0.0884 |
| Week 24 | 12 | 5.62 ± 14.63 | 0.3223 | 14 | 4.85 ± 7.46 | 0.0034 |
| Physical Functioning |  |  |  |  |  |  |
| Week 12 | 12 | 3.91 ± 8.85 | 0.1953 | 14 | 2.90 ± 12.88 | 0.6113 |
| Week 24 | 12 | 7.81 ±15.40 | 0.1348 | 14 | 4.02 ± 9.68 | 0.2500 |
| Emotional Functioning |  |  |  |  |  |  |
| Week 12 | 12 | 4.17 ± 12.58 | 0.3125 | 14 | 0.71 ± 8.05 | 1.0000 |
| Week 24 | 12 | 4.58 ± 24.54 | 0.6172 | 14 | 1.79 ± 9.53 | 0.6250 |
| Social Functioning |  |  |  |  |  |  |
| Week 12 | 11 | 0.00 ± 10.00 | 1.0000 | 14 | 6.43 ± 12.16 | 0.1250 |
| Week 24 | 11 | 3.18 ± 9.82 | 0.3438 | 14 | 6.43 ± 10.82 | 0.1250 |
| School Functioning |  |  |  |  |  |  |
| Week 12 | 10 | 8.50 ± 14.54 | 0.1250 | 14 | 5.36 ± 13.51 | 0.1250 |
| Week 24 | 10 | 7.67 ± 15.30 | 0.1875 | 14 | 7.14 ± 14.24 | 0.0625 |
| PedsQL (2-18 years old, parent proxy report) |  |  |  |  |  |  |
| Total Score |  |  |  |  |  |  |
| Week 12 | 10 | 1.72 ± 9.69 | 0.8203 | 12 | -3.24 ± 13.24 | 0.5635 |
| Week 24 | 10 | 2.83 ± 13.04 | 0.668 | 12 | 0.40 ± 10.94 | 0.7739 |
| Physical Functioning |  |  |  |  |  |  |
| Week 12 | 10 | 1.56 ± 7.69 | 0.8125 | 12 | -4.17 ± 15.44 | 0.4688 |
| Week 24 | 10 | 3.13 ± 13.01 | 0.7617 | 12 | 2.08 ± 13.74 | 0.5547 |
| Emotional Functioning |  |  |  |  |  |  |
| Week 12 | 10 | -1.00 ± 13.29 | 1.0000 | 12 | -0.83 ± 16.21 | 1.0000 |
| Week 24 | 10 | -1.00 ± 16.12 | 0.9609 | 12 | 0.42 ± 11.57 | 0.9922 |
| Social Functioning |  |  |  |  |  |  |
| Week 12 | 9 | -1.67 ± 9.35 | 0.7500 | 12 | -5.83 ± 14.28 | 0.1875 |
| Week 24 | 9 | 0.56 ± 10.44 | 1.0000 | 12 | -1.67 ± 14.67 | 0.8750 |
| School Functioning |  |  |  |  |  |  |
| Week 12 | 8 | 10.63 ±18.41 | 0.1875 | 12 | -2.08 ±16.85 | 0.8203 |
| Week 24 | 8 | 11.46 ± 19.89 | 0.1875 | 12 | -0.83 ± 13.11 | 0.7969 |
| SF-36 (≥ 26 years old) |  |  |  |  |  |  |
| Physical Component Summary |  |  |  |  |  |  |
| Week 12 | 5 | 0.16 ± 2.35 | 1.0000 | 2 | 3.65 |  |
| Week 24 | 5 | -0.76 ± 1.62 | 0.6250 | 2 | 14.6 |  |
| Mental Component Summary |  |  |  |  |  |  |
| Week 12 | 5 | -2.32 ± 5.50 | 0.4375 | 2 | -7.65 |  |
| Week 24 | 5 | 0.32 ± 5.91 | 1.0000 | 2 | -16.45 |  |
| Role Component Summary |  |  |  |  |  |  |
| Week 12 | 5 | 0.30 ± 2.73 | 0.8750 | 2 | -7.6 |  |
| Week 24 | 5 | -1.02 ± 3.44 | 0.4375 | 2 | -12.00 |  |
| Changes in Performance Status |  |  |  |  |  |  |
| Lansky play-performance scale |  |  |  |  |  |  |
| Week 12 | 8 | 0.0 ± 0.0 | - | 10 | 0.0 ± 0.0 | - |
| Week 24 | 8 | 1.3 ± 3.5 | 0.3506 | 10 | 1.0 ± 3.2 | 0.3434 |
| Kranofsky Performance Status |  |  |  |  |  |  |
| Week 12 | 9 | 3.3 ± 7.1 | 0.1950 | 7 | 1.4 ± 3.8 | 0.3559 |
| Week 24 | 9 | 3.3 ± 7.1 | 0.1950 | 7 | 1.4 ± 3.8 | 0.3559 |

| Supplemental Table S2. Changes in Lesion Volume | | | | | | | |
| --- | --- | --- | --- | --- | --- | --- | --- |
| Group | Patient ID | Disease  Diagnosis | MRI volumetry (cm^3^) | | | % change from baseline | |
|  |  |  | Baseline | Week 12 | Week 24 | Week 12 | Week 24 |
| 50mg | 1 | KTS | 761.95 | 743.01 | 981.23 | -2.5 | 28.8 |
|  | 2 | VM | 144.57 | 131.83 | 120.09 | -8.8 | -16.9 |
|  | 3 | VM | 134.51 | 113.63 | 132.36 | -15.5 | -1.6 |
|  | 7 | VM | 333.63 | 306.02 | 363.46 | -8.3 | 8.9 |
|  | 11 | KTS | 2683.67 | 2755.59 | 2396.82 | 2.7 | -10.7 |
|  | 12 | LM | 380.59 | 382.00 | 286.76 | 0.4 | -24.7 |
|  | 13 | VM | 103.31 | 102.81 | 107.02 | -0.5 | 3.6 |
|  | 19 | VM | 17.39 | 22.17 | 21.89 | 27.5 | 25.9 |
|  | 20 | KTS | 2141.12 | 1901.12 | 1692.99 | -11.2 | -20.9 |
|  | 22 | LM | 4.74 | 4.47 | 3.86 | -5.7 | -18.6 |
|  | 24 | VM | 136.83 | 121.08 | 120.27 | -11.5 | -12.1 |
|  | 27 | VM | 30.24 | 36.38 | 30.40 | 20.3 | 0.5 |
|  | 28 | VM | 44.16 | 39.29 | 44.67 | -11.0 | 1.2 |
|  | 29 | KST | 1302.84 | 1046.26 | 1006.22 | -19.7 | -22.8 |
|  | 32 | KTS | 899.23 | 820.22 | 711.22 | -8.8 | -20.9 |
|  | 34 | KTS | 834.23 | 1070.67 | 1349.89 | 28.3 | 61.8 |
|  | 35 | VM | 3710.41 | 3240.67 | 2917.93 | -12.7 | -21.4 |
| 100mg | 4 | KTS | 966.96 | 609.41 | 528.71 | -37.0 | -45.3 |
|  | 5 | VM | 291.44 | 269.35 | 262.93 | -7.6 | -9.8 |
|  | 6 | LM | 4242.53 | 4057.82 | 4071.76 | -4.4 | -4.0 |
|  | 8 | VM | 1557.22 | 1899.46 | 1624.29 | 22.0 | 4.3 |
|  | 9 | KTS | 236.86 | 251.24 | 235.97 | 6.1 | -0.4 |
|  | 10 | KTS | 442.19 | 205.49 | 213.70 | -53.5 | -51.7 |
|  | 14 | VM | 29.49 | 48.87 | 46.77 | 65.7 | 58.6 |
|  | 15 | VM | 215.73 | 232.38 | 194.07 | 7.7 | -10.0 |
|  | 16** | LM | 209.94 | 211.10 | n.d. | 0.6 | 0.6 |
|  | 17 | VM | 82.13 | 54.44 | 53.86 | -33.7 | -34.4 |
|  | 18 | KTS | 894.83 | 925.12 | 899.66 | 3.4 | 0.5 |
|  | 21* | KTS | 425.11 | n.d. | n.d. | n.d. | n.d. |
|  | 23 | VM | 172.39 | 153.33 | 123.26 | -11.1 | -28.5 |
|  | 25 | VM | 90.77 | 80.41 | 74.78 | -11.4 | -17.6 |
|  | 26 | LM | 19.20 | 18.20 | 16.39 | -5.2 | -14.7 |
|  | 30 | KTS | 359.44 | 352.25 | 260.28 | -2.0 | -27.6 |
|  | 31 | VM | 1639.80 | 1676.12 | 1499.08 | 2.2 | -8.6 |
|  | 33 | KTS | 1073.23 | 1003.70 | 797.59 | -6.5 | -25.7 |

* ART-001 treatment was discontinued

** Because of infection at the target lesion, the MRI scan at Day 169 was not performed and the last observation carried forward was applied.

n.d., not determined

| **Supplemental Table S3. TEAEs** | | | |
| --- | --- | --- | --- |
|  |  | **ART-001, 50 mg (n=17)** | **ART-001, 100 mg (n=18)** |
| **Gastrointestinal disorders** | | 4 (23.5) | 7 (38.9) |
|  | Abdominal pain | 0 (0.0) | 1 (5.6) |
|  | Angular cheilitis | 1 (5.9) | 1 (5.6) |
|  | Diarrhea | 2 (11.8) | 2 (11.1) |
|  | Gastric ulcer | 1 (5.9) | 0 (0.0) |
|  | Hemorrhoids | 0 (0.0) | 1 (5.6) |
|  | Nausea | 2 (11.8) | 3 (16.7) |
|  | Stomatitis | 0 (0.0) | 2 (11.1) |
|  | Vomiting | 0 (0.0) | 2 (11.1) |
| **General disorders and administration site conditions** | | 3 (17.6) | 0 (0.0) |
|  | Pyrexia | 3 (17.6) | 0 (0.0) |
| **Infections and infestations** | | 7 (41.2) | 6 (33.3) |
|  | Eye infection | 0 (0.0) | 1 (5.6) |
|  | Hordeolum | 0 (0.0) | 1 (5.6) |
|  | Infection | 0 (0.0) | 1 (5.6) |
|  | Nasopharyngitis | 0 (0.0) | 2 (11.1) |
|  | Otitis media | 1 (5.9) | 0 (0.0) |
|  | Pharyngitis | 1 (5.9) | 0 (0.0) |
|  | Upper respiratory tract infection | 2 (11.8) | 2 (11.1) |
|  | Abscess limb | 1 (5.9) | 0 (0.0) |
|  | Enteritis infectious | 1(5.9) | 0 (0.0) |
|  | Systemic viral infection | 1 (5.9) | 0 (0.0) |
|  | COVID-19 pneumonia | 3 (17.6) | 1 (5.6) |
| **Injury, poisoning and procedural complications** | | 2 (11.8) | 2(11.1) |
|  | Fall | 1 (5.9) | 0 (0.0) |
|  | Fracture | 0 (0.0) | 1 (5.6) |
|  | Wound secretion | 1(5.9) | 0 (0.0) |
|  | Mouth injury | 0(0.0) | 1 (5.6) |
|  | Skin abrasion | 1 (5.9) | 0 (0.0) |
| **Investigations** | | 6 (35.3) | 4 (22.2) |
|  | Alanine aminotransferase increased | 2 (11.8) | 3 (16.7) |
|  | Aspartate aminotransferase increased | 2 (11.8) | 3 (16.7) |
|  | Beta 2 microglobulin urine increased | 2 (11.8) | 2 (11.1) |
|  | Blood bilirubin increased | 1 (5.9) | 0 (0.0) |
|  | C-reactive protein increased | 1 (5.9) | 0 (0.0) |
|  | Urine ketone body present | 1 (5.9) | 0 (0.0) |
|  | Neutrophil gelatinase-associated lipocalin increased | 0 (0.0) | 1 (5.6) |
| **Metabolism and nutrition disorders** | | 0 (0.0) | 1 (5.6) |
|  | Decreased appetite | 0 (0.0) | 1 (5.6) |
| **Musculoskeletal and connective tissue disorders** | | 1 (5.9) | 1 (5.6) |
|  | Myalgia | 1 (5.9) | 0 (0.0) |
|  | Pain in extremity | 0 (0.0) | 1 (5.6) |
| **Nervous system disorders** | | 0 (0.0) | 2 (11.1) |
|  | Hyperesthesia | 0 (0.0) | 1 (5.6) |
|  | Somnolence | 0 (0.0) | 1 (5.6) |
| **Renal and urinary disorders** | | 2 (11.8) | 0 (0.0) |
|  | Hematuria | 1 (5.9) | 0 (0.0) |
|  | Nephropathy toxic | 1 (5.9) | 0 (0.0) |
| **Respiratory, thoracic and mediastinal disorders** | | 3 (17.6) | 1 (5.6) |
|  | Cough | 1 (5.9) | 0 (0.0) |
|  | Epistaxis | 1 (5.9) | 0 (0.0) |
|  | Hypoxia | 0 (0.0) | 1 (5.6) |
|  | Rhinorrhea | 1 (5.9) | 0 (0.0) |
| **Skin and subcutaneous tissue disorders** | | 3(17.6) | 3 (16.7) |
|  | Acne | 1 (5.9) | 1 (5.6) |
|  | Eczema | 0 (0.0) | 1 (5.6) |
|  | Hyperkeratosis | 0 (0.0) | 1 (5.6) |
|  | Rash | 1 (5.9) | 0 (0.0) |
|  | Skin ulcer | 1 (5.9) | 0 (0.0) |
| **Vascular disorders** | | 3 (17.6) | 1 (5.6) |
|  | Lymphorrhea | 2 (11.8) | 0 (0.0) |
|  | Hemorrhage | 1 (5.9) | 0 (0.0) |
|  | Internal hemorrhage | 0 (0.0) | 1 (5.6) |
